# Quality attributes and cooking properties of commercial Thai rice noodles

**DOI:** 10.7717/peerj.11113

**Published:** 2021-04-06

**Authors:** Supaluck Kraithong, Saroat Rawdkuen

**Affiliations:** Unit of Innovative Food Packaging and Biomaterials, School of Agro-Industry, Mae Fah Luang University, Muang Chiang Rai, Thailand

**Keywords:** Accepatability, Cooking properties, Pasting, Rice noodles, Sensory properties

## Abstract

One of the most popular and abundant traditional foods in Asian countries is dried rice noodles. In fact, the demand for this product has increased steadily around the world in recent years. The qualities of rice noodles are directly related to the specific preferences of consumers. Hence, the present study aimed to determine the properties of eight commercial dried rice noodles that are readily available in most Thai markets. The specific properties under investigation and comparison in this study were proximate composition, amylose content, color, pasting quality, cooking quality, texture, and sensory properties. The specimens were divided into two groups: white (A, B, C, D, and E) and colored rice noodles (F, G, and H). The results showed that the proximate composition, amylose content, and color of both white and colored rice noodles were significantly different (*p* < 0.05). The lowest cooking losses in white and colored rice noodles were 0.11% (B) and 2.03% (G) (*p* < 0.05), respectively. Higher values of pasting (setback and final viscosities) and texture properties (tensile strength and extensibility) provided higher overall acceptability. The highest scores for acceptability of white and colored rice noodles were 7.00 (B) and 5.87 (H) (*p* < 0.05), respectively.

## Introduction

Dried rice noodles are one of the most popular traditional foods in Asian countries. Moreover, their popularity has spread, and they are readily consumed in over 80 countries worldwide (Tong et al., 2015). Consequently, the rice noodle industry in Thailand has been booming to meet this growing demand (Purwandari et al., 2014). Rice noodles are generally produced with high- amylose white rice flour (>25%) (Hsu et al., 2015). Recently, higher levels of nutrients (e.g., protein, fiber) and bioactive compounds (e.g., anthocyanin, proanthocyanidin) have been found in colored/pigmented rice flour (Pereira-Caro et al., 2013). Accordingly, the rice noodle industry has been employing pigmented rice in rice noodle production to meet consumer demands for healthier alternatives (Sabbatini et al., 2014).

Noodle quality is highly dependent on the manufacturing processes and the initial quality of the raw material used in noodle production (rice flour) (Luo, Guo & Zhu, 2015). The time taken to steam and dry rice noodles affects their cooking properties and texture (Wang et al., 2016). The various qualities of rice flour (e.g., amylose content, chemical composition) are influenced by the rice variety used, which affects the texture and cooking properties of the resulting noodles (Chou, Yen & Li, 2014). There are many reports about the effects of ingredients and processes on rice noodle properties. *Sandhu, Maninder & Mukesh (2010)* studied the qualities of noodles made from blended potato and rice starches in ratios of 1:3, 1:1, and 3:1. It is discovered that noodles made from potato and rice starches in a 1:1 ratio had the lowest cooking time and cooked weight. *Huang & Lai (2010)* reported that noodles prepared from rice, wheat, and corn starches showed differences in texture properties. *Malahayati et al. (2017)* revealed that rice noodles prepared by steaming and boiling demonstrated differences in chemical and cooking properties. Basic information on rice noodle quality attribute is indispensable to Thai rice noodle factories who wish to further develop or create a new product with high acceptability and a large market share. However, the qualities of commercially available rice noodles found in typical Thai markets have been under-reported, resulting in limited industrial development of Thai rice noodles.

This work aims to determine the properties of rice noodles which are usually consumed in Thailand. The properties are texture, pasting, cooking, and sensorial properties including color. The results could be useful to rice noodle manufacturers who wish to improve the quality of their products in order to increase their share of the market and respond to consumers’ needs. In addition, the information provided will be useful for creating alternative products by substituting other functional ingredients while maintaining acceptable quality attributes.

## Materials and Methods

**Figure 1:**
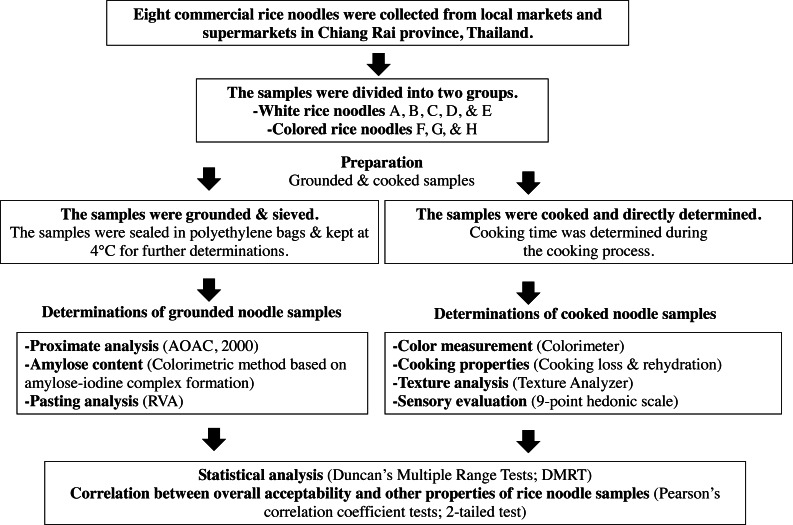
Schematic diagram of the research process.

The schematic diagram (Fig. 1) presents the overall process of this research, including sample collection, preparation steps, and evaluation of Thai commercial rice noodles.

### Samples and preparation

The noodle samples used in this work are available in the Thai market and generally consumed. The eight commercial dried rice noodles were purchased from local markets and supermarkets in Chiang Rai province, Thailand. The samples were divided into white (A, B, C, D, and E) and colored rice noodles (F, G, and H).

Commercial information provided for the noodle samples; sample A predominantly obtained from white rice (*Oryza sativa* L.) while sample B prepared from the combination of white rice with a small amount of wheat flour using an automatic machine. Sample C produced from 80% of white rice and 20% of tapioca starch. Sample D was claimed to be prepared by 100% of *O. sativa* L. The main ingredient of sample E was Phitsanulok white rice. Noodle sample F, G, and H were mainly prepared from colored rice varieties which are Hom Nin (black rice), Brown Khao Dawk Mali 105 (brown rice), and Hom Mali Dang (red rice), respectively.

The samples were ground in a blender (HR2001, Philips, China) for 5 min (pausing every 1 min) and then passed through a 60-mesh sieve. The ground samples were sealed in polyethylene bags and kept at 4 °C for further determinations.

### Proximate composition

The moisture (method 934.01), ash (method 945.46), crude protein (N ×5.95) (method 992.15 (39.1.16); the Kjeldahl method), crude fat (method 954.02; the Soxhlet method), and crude fiber contents (method 978.10; using automatic crude fiber analysis) of the samples were determined according to the *AOAC (2000)*. The carbohydrate content was calculated by subtracting the total percentage of the other components from 100%.

### Amylose content

The amylose content was examined according to the method of *Juliano (1971)*. The ground samples (100 mg) were mixed with 95% ethanol (1 mL) and 2 M NaOH (9 mL) and their volumes were adjusted to 100 mL with distilled water. The mixture was combined with 0.2% iodine solution (2 mL), and the absorbance was measured at 620 nm wavelength (Genesy 10S UV-Vis spectrophotometer, Thermo Scientific, USA). The amylose content was estimated based on a standard curve prepared with potato starch; the concentrations of amylose solutions were 0%–40% (dry weight basis) with the correlation coefficient (R^2^) of 0.9993.

### Color measurement

Color parameters in terms of L* (Lightness), a* (Redness), and b* (Yellowness) were determined by using colorimeter (Miniscan EZ, USA) which calibrated before determination.

### Cooking properties

The method of Wu et al. (2015) was used to evaluate cooking properties. Noodle strands (six cm in 5 g) were cooked in 150 ml boiling distilled water. The optimal cooking time was determined as the time at which the noodle core was no longer visible. Observation of the noodle core, a rice noodle strand was sampling and then squeezed between the two glass plates every 30 s.

Cooking loss and rehydration were determined according to the method of *Wu et al. (2015)*. The cooked rice noodles were rinsed with distilled water then the water obtained were collected and dried at 105 °C until constant weights were achieved. Cooking loss and rehydration were calculated using equation Eqs. (1) and (2):

(1) \begin{eqnarray*}\text{Cooking loss}(\text{%})= \frac{\text{Weight of dry matter in cooking water (g)}}{\text{Weight of dry noodles(g)}} \times 100\end{eqnarray*}

(2) \begin{eqnarray*}\text{Rehydration}(\text{%})=\nonumber\\\displaystyle \frac{\text{Weight of cooked noodles (g) - Weight of uncooked noodles (g)}}{\text{Weight of uncooked noodles (g)}} \times 100\end{eqnarray*}

### Pasting properties

Pasting properties were measured with a Rapid Visco Analyser (RVA 4500, Perten Instruments, Sweden). The powdered samples (approximately 3 g) were weighed in a canister and then combined with distilled water (approximately 25 mL). The noodle suspensions were subjected to pasting analysis. The results were obtained under the following conditions: the temperature was maintained at 60 °C for 2 min and then raised to 95 °C within 6 min, maintained for 4 min, cooled to 50 °C and held for 4 min. RVA parameters including peak, trough, breakdown, final, and setback viscosities were recorded after the determination.

### Textural properties

Texture properties were measured with a texture analyzer (model TA. XT. Plus, Stable MicroSystems Ltd., England) based on the method of *Ye & Sui (2016).* Rice noodles were cooked for their optimal cooking times. The noodle strands were compressed with a hemispherical probe (P/0.5HS) at a test speed of 2.0 mm/s with 50% strain. Then, hardness (g), adhesiveness (gsec), cohesiveness (no unit), gumminess (g), springiness (no unit), and chewiness (gmm) were obtained. Tensile strength (g) and extensibility (mm) were examined with a pair of spaghetti/noodles tensile grips at a cross head velocity of 3.0 mm/s.

### Sensory evaluation

Sensory evaluation was performed based on the method of *Purwandari et al. (2014)*. The samples were cooked for the optimal cooking times and immediately served with chicken soup (1:2 g/g) for 30 untrained panelists; most panelists were students and staff of Mae Fah Luang University. Evaluation of sensory properties in terms of color, flavor, taste, softness, stickiness, elasticity, and overall acceptability were carried out by using a 9-point hedonic scale, where 9 = extremely like and 1 = extremely dislike. Evaluations of flavor, taste, softness, stickiness, and elasticity were performed under a dim red light in order to avoid possible prejudice due to differences in noodle color.

### Statistical analysis

An analysis of variance (ANOVA) was completed. Comparisons between means were carried out using Duncan’s Multiple Range Tests (DMRT). All determinations were conducted in triplicate except for texture analysis, which was performed in 6 replications, and sensory evaluation, which was completed in 30 replications. Correlations between overall acceptability and other properties of rice noodle samples were analyzed using Pearson’s correlation coefficient tests (2-tailed test). Differences were considered significant at *p* < 0.05. The analysis was performed by using an SPSS package (SPSS 17.0 for window, SPSS Inc, Chicago, IL).

## Results

### Proximate composition and amylose content

The chemical composition of Thai rice noodles is shown in Table 1. Differences in the moisture (10.03%–11.75%), ash (0.42%–0.94%), crude fat (0.63%–0.99%), crude fiber (0.40%–0.81%), crude protein (4.04%–6.55%), and carbohydrate contents (80.03%–83.22%) of white rice noodles were observed (*p* < 0.05). The moisture (11.51%–11.81%) and crude fiber contents (1.29%–1.47%) among the colored rice noodle samples were not significantly different (*p* > 0.05). However, the levels of other constituents of colored noodle samples were considerably different (*p* < 0.05).

**Table 1:** Proximate composition and amylose content of commercial rice noodles.

Rice noodles	Moisture (%)	Ash (%)	Crude Fat (%)	Crude Fiber (%)	Crude Protein (%)	Carbohydrate (%)	Amylose (%)
White							
A	11.13 ±0.76^a^	0.94 ±0.04^a^	0.82 ±0.04^b^	0.81 ±0.04^a^	6.55 ±0.92^a^	80.15 ±0.15^b^	22.18 ±0.26^d^
B	11.75 ±0.76^a^	0.50 ±0.02^b^	0.64 ±0.12^d^	0.65 ±0.04^b^	6.43 ±0.38^a^	80.03 ±0.56^b^	23.36 ±0.33^c^
C	10.03 ±0.73^b^	0.58 ±0.10^b^	0.63 ±0.06^d^	0.59 ±0.04^c^	4.04 ±0.38^b^	83.22 ±1.01^a^	24.61 ±0.44^b^
D	11.23 ±0.82^a^	0.84 ±0.04^a^	0.99 ±0.02^a^	0.43 ±0.06^d^	4.71 ±0.76^b^	82.40 ±0.60^a^	24.80 ±0.30^b^
E	11.34 ±0.63^a^	0.42 ±0.04^c^	0.71 ±0.05^c^	0.40 ±0.05^d^	6.36 ±0.42^a^	80.60 ±0.43^b^	27.24 ±0.50^a^
Color							
F	11.81 ±0.12^A^	0.50 ±0.03^B^	3.63 ±0.19^A^	1.47 ±0.18^A^	7.36 ±0.32^A^	75.13 ±1.01^C^	19.74 ±0.34^A^
G	11.68 ±0.20^A^	0.90 ±0.03^A^	2.91 ±0.27^B^	1.29 ±0.12^AB^	6.98 ±0.26^AB^	77.18 ±0.37^A^	17.61 ±0.26^C^
H	11.51 ±0.26^A^	0.56 ±0.02^B^	2.65 ±0.33^B^	1.36 ±0.05^AB^	6.55 ±0.60^B^	76.43 ±0.52^B^	18.28 ±0.28^B^

**Notes.**

Means of triplicates ± standard deviation. ^a– d, A–C^ Superscript letters for white and colored rice noodles, the same superscript letters in a column are not significantly different at *p* < 0.05 level.

The amylose content was 22.18%–27.24% in white rice noodle samples and 17.61%–19.74% in colored rice noodles, as shown in Table 1. The lowest amylose content in white and colored rice noodles was 22.18% (A) and 17.61% (G) (*p* > 0.05), respectively.

### Color attributes

The L* value of white rice noodles varied from 43.03 to 51.01, whereas, the a* and b* values were in the range from -0.86 to −0.58 and from 1.59 to 7.09 (Table 2), respectively. The highest L*, a*, and b* values in white rice noodles were 51.01 (E), −0.58 (D), and 7.09 (E) (*p* < 0.05), respectively.

**Table 2:** Color attributes and cooking properties of commercial rice noodles.

Rice noodles	Color			Cooking properties		
	L*	a*	b*	Cooking time (min)	Cooking loss (%)	Rehydration (%)
White						
A	46.87 ±1.66^b^	−0.86 ±0.09^d^	2.60 ±0.39^d^	3.00 ±0.50^b^	0.33 ±0.07^c^	124.09 ±2.97^*bc*^
B	44.30 ±1.17^*bc*^	−0.76 ±0.18^c^	6.66 ±0.52^b^	4.33 ±0.29^a^	0.11 ±0.02^c^	142.88 ±2.03^b^
C	46.78 ±0.94^b^	−0.67 ±0.20^b^	1.59 ±0.43^*e*^	4.17 ±0.29^a^	3.02 ±0.07^a^	115.96 ±2.62^c^
D	43.03 ±0.08^*bc*^	−0.58 ±0.10^a^	5.03 ±0.07^c^	3.17 ±0.29^b^	1.52 ±0.31^b^	110.04 ±2.97^c^
E	51.01 ±1.14^a^	−0.66 ±0.04^b^	7.09 ±0.14^a^	4.50 ±0.50^a^	0.39 ±0.09^c^	157.42 ±2.10^a^
Color						
F	28.07 ±1.39^B^	3.11 ±0.21^B^	0.25 ±0.05^C^	7.17 ±0.29^B^	3.04 ±0.33^A^	222.57 ±1.04^A^
G	44.94 ±0.13^A^	2.23 ±0.39^C^	12.38 ±0.42^A^	7.33 ±0.29^B^	2.03 ±0.16^B^	189.93 ±0.18^B^
H	23.02 ±0.52^C^	7.95 ±0.08^A^	7.33 ±0.30^B^	8.50 ±0.50^A^	2.48 ±0.46^B^	189. 93 ±1.20^B^

**Notes.**

Means of triplicates ± standard deviation. ^a-e, A–C^ Superscript letters for white and colored rice noodles, the same superscript letters in a column are not significantly different at *p* < 0.05 level.

The L*, a*, and b* values of colored rice noodles were 23.02–44.94, 2.23–7.95, and 0.25–12.38, respectively. The highest L* (44.94), a* (7.95), and b* (12.38) values of pigmented rice noodles were found in samples G, H, and G (*p* < 0.05) (Table 2), respectively. The higher a* and b* values could be caused by the pigment of the samples such as anthocyanin and proanthocyanidin which can benefit consumer health (Pereira-Caro et al., 2013).

### Cooking time

The cooking times of white (3.00–4.50 min) and colored rice noodles (7.17–8.50 min) are shown in Table 2. The lowest cooking times in white (3.00 min) and colored rice noodles (7.17 min) were found in samples D and F (*p* < 0.05) (Table 2), respectively, whereas, sample E (white rice noodle) and H (colored rice noodle) showed the highest cooking times of 4.50 min and 8.50 min (*p* < 0.05), respectively.

### Cooking loss

Cooking loss indicates the amounts of solid components that leach out from the noodle structure during cooking. Cooking loss values of white and colored rice noodles were 0.11%–3.02% and 2.03%–3.04% (Table 2), respectively. The results showed that the lowest cooking losses from white (B) and colored rice noodles (G) were 0.11% and 2.03% (*p* < 0.05) (Table 2), respectively. Whereas, the highest cooking loss values in white (C) and colored rice noodle (F) samples were 3.02% and 3.04%, respectively.

### Rehydration

The percentages of rehydration varied from 110.04% to 157.42% in white rice noodles and from 189.93% to 222.57% in colored rice noodles (Table 2). The highest rehydration values in white (E) and colored rice noodles (F) were 157.42% and 222.57% (*p* < 0.05) (Table 2), respectively, representing a high capacity to absorb water during the cooking process. The lowest rehydration value in the white rice sample was found at 110.04% (D) while it was 189.93% in colored rice noodles (G and H).

### Pasting properties

**Figure 2:**
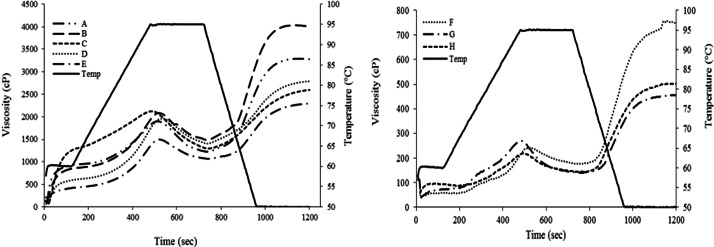
RVA profiles of commercial Thai rice noodles; white (A) and colored rice noodles (B).

The pasting properties of white and colored Thai rice noodle samples varied as indicated in the RVA profile (Fig. 2). In white rice noodles, the peak, trough, breakdown, final, and setback viscosities were 1,519.2–2,082.0 cP, 1,082.4–1,501.0 cP, 449.6–810.0 cP, 2,311.4–4,009.2 cP, and 1,126.0–2,512.8 cP, respectively. In colored rice noodles, the peak viscosity was 212.0–270.4 cP, trough viscosity was 140.0–151.4 cP, breakdown viscosity was 57.8–129.6 cP, final viscosity was 455.0–774.6 cP, and setback viscosity was 315.0–504.4 cP (Table 3).

**Table 3:** Pasting properties of commercial rice noodles.

Rice noodles	Peak viscosity (cP)	Trough viscosity (cP)	Breakdown (cP)	Final viscosity (cP)	Setback (cP)
White					
A	1,951.6 ±43.80^b^	1,244.4 ±20.79^b^	700.2 ±9.27^b^	3,386.8 ±61.00^b^	2,108.0 ±51.18^b^
B	2,038.6 ±33.62^a^	1,501.0 ±15.80^a^	589.4 ±9.53^c^	4,009.2 ±17.14^a^	2,512.8 ±40.75^a^
C	2,082.0 ±20.97^a^	1,270.0 ±13.21^b^	810.0 ±8.69^a^	2,586.8 ±20.35^c^	1,302.2 ±14.36^c^
D	1,911.2 ±18.35^b^	1,265.2 ±19.85^b^	449.6 ±7.37^d^	2,606.4 ±54.98^c^	1,126.0 ±18.31^d^
H	1,519.2 ±26.48^c^	1,082.4 ±14.93^c^	455.4 ±5.80^d^	2,311.4 ±16.32^d^	1,264.4 ±14.70^c^
Color					
E	212.0 ±5.13^C^	151.4 ±5.60^A^	57.8 ±6.36^C^	774.6 ±26.41^A^	504.4 ±18.96^A^
F	270.4 ±5.51^A^	140.0 ±7.29^B^	129.6 ±4.79^A^	455.0 ±40.10^C^	315.0 ±19.21^C^
G	230.4 ±3.75^B^	148.6 ±6.73^B^	79.2 ±6.21^B^	528.6 ±11.30^B^	371.8 ±27.58^B^

**Notes.**

Means of triplication ± standard deviation. ^a–d, A–C^ Superscript letters for white and colored rice noodles, the same superscript letters in a column are not significantly different at *p* < 0.05 level.

In white rice noodles, sample B presented high values of peak (2,038.6 cP), trough (1,501.0 cP), final (4,009.2 cP), and setback viscosities (2,512.8 cP) but low breakdown viscosity (589.4 cP) (*p* < 0.05), as shown in Table 3. This is considered as a desirable characteristic; a required property of rice noodle such as soft texture with strong/flexible structure and high shear force resistance could be obtained from the high peak, final, and setback viscosities and with low breakdown (Wang et al., 2016; Chung, Cho & Lim, 2012; Yadav, Yadav & Kumar, 2011). In contrast, none of colored rice noodle samples showed favorable attributes.

### Texture properties

Texture properties of Thai rice noodles were shown in Table 4. In both white and colored rice noodles, no difference in springiness was observed (*p* > 0.05) while other TPA parameters were significantly different (*p* < 0.05). High tensile strength and extensibility are required characteristics for noodles since they indicate the strong structure and high cooking tolerance (Malahayati et al., 2017; Nura et al., 2011). In white rice noodles, samples B and A represented the highest tensile strength (124.83 g) and extensibility (30.72 mm) (*p* < 0.05), respectively. In colored rice noodles, sample G showed the highest values of both parameters; tensile strength was 62.19 g, and extensibility was 25.54 mm (Table 4) (*p* < 0.05).

**Table 4:** Texture properties of commercial rice noodles.

Rice noodles	Tensile strength (g)	Extensibility (mm)	Hardness (g)	Adhesiveness (gsec)	Cohesiveness	Gumminess	Springiness	Chewiness (gmm)
White								
A	49.97 ±1.90^c^	30.72 ±2.79^a^	2042.1 ±24.87^b^	−15.04 ±1.48^c^	0.80 ±0.09^b^	725.51 ±18.16^a^	0.85 ±0.04^a^	719.69 ±22.13^b^
B	124.83 ±8.53^a^	27.26 ±2.65^a^	1137.84 ±26.14^c^	−15.64 ±2.53^c^	0.60 ±0.07^d^	638.56 ±31.13^b^	0.85 ±0.06^a^	636.11 ±22.86^c^
C	66.30 ±3.29^b^	23.22 ±2.92^b^	927.39 ±26.02^d^	−27.66 ±2.03^b^	0.62 ±0.03^d^	573.09 ±9.57^c^	0.81 ±0.03^a^	568.26 ±12.26^d^
D	16.11 ±2.89^d^	14.37 ±3.31^c^	981.40 ±15.98^d^	−32.42 ±2.42^a^	0.77 ±0.06^c^	261.73 ±11.40^d^	0.80 ±0.02^a^	260.64 ±8.70^*e*^
E	40.47 ±2.29^c^	29.73 ±3.82^a^	3985.52 ±40.78^a^	−6.23 ±1.42^*d*^	1.35 ±0.20^a^	751.99 ±34.28^a^	0.84 ±0.05^a^	1219.41 ±22.14^a^
Color								
F	19.02 ±2.30^B^	16.84 ±3.41^B^	4837.71 ±40.56^B^	−10.48 ±1.64^A^	0.41 ±0.07^B^	831.05 ±27.29^B^	0.85 ±0.04^A^	1525.32 ±19.59^C^
G	62.19 ±2.01^A^	25.54 ±2.28^A^	5020.80 ±19.41^*AB*^	−6.41 ±1.84^B^	0.95 ±0.07^A^	865.22 ±29.87^B^	0.85 ±0.09^A^	2095.56 ±10.90^B^
H	14.11 ±3.10^*BC*^	14.53 ±2.23^B^	5292.61 ±61.43^A^	−6.35 ±1.00^B^	0.12 ±0.03^C^	952.86 ±38.73^A^	0.83 ±0.08^A^	3867.00 ±67.03^A^

**Notes.**

^a–e, A–C^ Superscript letters for white and colored rice noodles, the same superscript letters are not significantly different at *p* < 0.05 level (*n* = 6). Adhesiveness value; the more negative value indicates the higher adhesiveness.

Higher hardness (3,985.52 g) in sample E resulted in the higher gumminess (751.99) and chewiness (1219.41 gmm) (*p* < 0.05) compared with other white rice noodle samples (Table 4). On the other hand, lower hardness led to higher adhesiveness and lower cohesiveness in other white noodle samples.

Colored rice noodles showed a similar tendency to white rice noodles. The highest hardness (5,292.61 g) in sample H resulted in the highest gumminess (952.86) and chewiness (3,867.00 gmm) (*p* < 0.05). In contrast, sample F presented the lowest hardness (4,837.71 g) and cohesiveness (0.41) (*p* < 0.05) but exhibited the highest adhesiveness (−10.48 gsec) (*p* < 0.05) (Table 4); the more negative value refers to the greater adhesiveness.

### Sensory evaluation

Differences were observed in the color (6.67–7.20), flavor (5.77–6.87), taste (5.17–6.77), softness (4.60–7.17), stickiness (5.07–6.33), elasticity (4.57–6.23), and overall acceptability scores (5.30–7.00) of commercial white rice noodles (*p* < 0.05) (Table 5). However, sensory scores in terms of color, flavor, taste, and stickiness were not significantly different (*p* < 0.05) in colored rice noodles (Table 5). The highest overall acceptability score in white (7.00) and colored rice noodles (5.87) were found in samples B and H (*p* < 0.05) (Table 5), respectively. On the other hand, the lowest acceptability score (5.30) was found in samples E and G (*p* < 0.05) of white and colored rice noodles, respectively.

**Table 5:** Sensory scores of commercial (cooked) rice noodles.

Rice noodles	Sensory score						
	Color	Flavor	Taste	Softness	Stickiness	Elasticity	Overall acceptability
White							
A	7.07 ±1.11^a^	6.43 ±1.25^a^	6.63 ±1.38^a^	7.00 ±1.08^a^	6.33 ±1.12^a^	6.23 ±1.10^a^	6.87 ±1.22^a^
B	7.03 ±1.07^a^	6.87 ±1.33^a^	6.77 ±1.30^a^	7.17 ±1.32^a^	6.00 ±1.08^a^	5.73 ±0.83^*ab*^	7.00 ±1.14^a^
C	7.20 ±1.35^a^	6.17 ±1.15^*ab*^	6.13 ±1.04^a^	5.60 ±0.86^*bc*^	5.50 ±0.82^b^	5.27 ±0.45^*bc*^	6.03 ±0.81^*bc*^
D	7.10 ±1.21^a^	6.50 ±1.07^a^	6.40 ±1.13^a^	5.83 ±0.91^b^	6.27 ±1.08^a^	5.90 ±0.99^*ab*^	6.60 ±1.28^*ab*^
E	6.67 ±1.27^b^	5.77 ±1.01^b^	5.17 ±0.59^b^	4.60 ±0.56^c^	5.07 ±0.78^b^	4.57 ±0.50^c^	5.30 ±0.47^c^
Color							
F	5.33 ±0.48^A^	5.47 ±0.63^A^	5.50 ±0.82^A^	4.93 ±0.74^*AB*^	5.40 ±0.56^A^	4.93 ±0.83^*AB*^	5.73 ±0.78^A^
G	5.73 ±0.87^A^	5.33 ±0.55^A^	5.50 ±0.90^A^	4.37 ±0.49^B^	5.23 ±0.43^A^	4.50 ±0.51^B^	5.30 ±0.53^*AB*^
H	5.40 ±0.62^A^	5.83 ±1.02^A^	5.80 ±1.30^A^	5.20 ±0.48^A^	5.43 ±0.57^A^	5.20 ±0.55^A^	5.87 ±0.94^A^

**Notes.**

Sensory evaluation carried out by 9-point hedonic scale (9 = extremely like and 1 = extremely dislike, 30 untrained panelists). ^a–c, A–B^ superscript letters for white and colored rice noodles, the same superscript letters in a column are not significantly different at *p* < 0.05 level.

## Discussion

### Proximate composition and amylose content

The obtained results are in agreement with *Verma & Srivastav (2017)*, who reported the following composition of rice noodles prepared from aromatic and non-aromatic rice varieties which are Gopal Bhong, Govind Bhog, Badshah Bhog, Kalanamak, Swetganga, Khushboo, Sarbati, and Todal; moisture (8.90%–13.57%), ash (0.35%–0.73%), crude protein (6.87%–9.27%), crude fat (0.06%–0.92%), and carbohydrate (75.87%–82.70%).

The values of ash, crude fat, crude protein, and carbohydrate contents (Table 1) of colored rice noodles are similar to the findings of *Sompong et al. (2011)*. The authors reported that the values of ash, crude fat, crude protein, and carbohydrate contents in red and black rice varieties were 0.82%–1.74%, 1.15%–3.72%, 7.16%–10.85%, and 71.99%–79.27%, respectively. According to the Thai Industrial Standards Institute (Thai Industrial Standards Institute, 1990), the moisture content of dried rice noodles should be not higher than 12%. Thus, the moisture contents of all Thai noodle samples were in line with TISI standards. Higher contents of ash, crude fat, crude fiber, and crude protein in rice noodles generally promote higher nutrients that are desirable for consumers (Wang et al., 2016).

The amylose content of white rice noodles (Table 1) was similar to the findings of *Nura et al. (2011)*, which confirmed that rice noodles generally contained greater than 22% amylose. According to the work reported by Hsu et al. (2015), only white rice noodle sample E (27.24%) could have been made from high-amylose rice flour (>25%), whereas the amylose content of colored rice noodles was not different from that of *Ziegler et al. (2017)*, who reported an amylose content of 6.0%–23.3% for colored rice grains. All colored rice noodle samples could be prepared from low-amylose rice flour. The difference in amylose content among samples can lead to variation in noodle properties.

Generally, the chemical composition of rice noodles is influenced by rice varieties and growing conditions. Noodles prepared from colored rice varieties frequently present higher crude protein, crude fiber, and crude fat because of the higher outer layer remained (Pereira-Caro et al., 2013). Other ingredients in noodle formulation also affected some chemical compositions. For instance, the addition of protein source (e.g., wheat and pea) found to increase crude protein content in rice noodle whereas adding tapioca or cassava starch may cause the reduction of crude protein, crude fat, and crude fiber in the noodle product (Sofi et al., 2018; Pato et al., 2016). Rice noodle preparation methods (i.e., steaming, boiling) also show an effect on noodle composition. Boiling can lower the ash, crude fat, crude protein, and amylose content of rice noodles. This is because soluble nutrients leach out from the starch granules into the surrounding water during processing (Malahayati et al., 2017). During the drying process, the longer time and higher temperature result in lower moisture content (Han, Cho & Koh, 2011). Thus, differences in rice noodle processing methods between manufacturers led to variations in chemical composition between noodle samples.

### Color attributes

The variation in the color of white rice noodles can be influenced by their chemical composition and processing. Generally, a higher ash content in rice flour results in lower brightness in noodles (Sandhu, Maninder & Mukesh, 2010). In contrast, a high amylose content in rice flour contributes to high values of brightness (Yadav, Yadav & Kumar, 2011). Moreover, the drying process also affects noodle color because the longer duration and higher temperature of the process tends to reduce brightness and increase yellowness (Malahayati et al., 2017).

The differences in color among pigmented rice noodles could be influenced by their pigments. The higher values of a* and b* in colored rice noodles could be caused by proanthocyanidins and carotenoids, respectively (Pereira-Caro et al., 2013), whereas lower lightness is supported by anthocyanin in pigmented rice (Sabbatini et al., 2014). Our preliminary results also confirmed that all colored rice noodles contained total phenolic compounds (353.58–415.74 mg GAE/100g sample) as well as total anthocyanin pigments (0.61–1.71 cyanidin-3-glucoside equivalents, mg/L), whereas those compounds were not detected in all white rice noodle samples; the data are shown in the supplementary file. Thus, it can be concluded that those compounds can influence noodle color.

### Cooking time

The cooking times of white rice noodles (Table 2) were in agreement with the work of *Wandee et al. (2015)*, which reported a cooking time of 3.0–4.5 min. A short cooking time is indicative of a good quality rice noodle (Hormdok & Noomhorm, 2007). High fiber content has been found to shorten the cooking time in rice noodles because it promotes water absorption by rice noodles (Tong et al., 2015). In white rice noodles, sample E presented the longest cooking time (4.50 min) (*p* < 0.05). This could be due to the highest amylose content present in this sample (*p* < 0.05). After noodle processing, amylose presents a high degree of retrogradation, resulting in a rigid structure of the noodle sample, which leads to a longer cooking time (Chou, Yen & Li, 2014). In colored rice noodles, a longer cooking time could be encouraged by high contents of proteins and lipids in rice noodles, in which the contents interact with amylose during noodle processing, interrupting water absorption (Hsu et al., 2015).

### Cooking loss

Cooking loss indicates the amounts of the solid components that leach out from the noodle structure during cooking. *Chung, Cho & Lim (2012)* and *Sangpring, Fukuoka & Ratanasumawong (2015)* confirmed that cooking loss in rice noodles was around 0.41%–5.11%. Low cooking loss is a desirable property of rice noodles. It indicates the ability to maintain the structural integrity of the noodle during cooking (Ahmed, Qazi & Jamal, 2015). Generally, high amylose content contributes to low cooking losses by providing structural strength to noodles by the junction zones or three-dimensional networks, reducing solid loss (Wang et al., 2016). The appropriate number of this content has been reported at 20%–25%; the lower and higher amounts are not suitable for noodle production causing the weaker and very rigid structures which are easy to be broken down, respectively (Xuan et al., 2020). Sample C and D exhibited higher cooking loss compared to other white rice samples even containing the proper amylose content with lower fiber content (Table 2) (*p* < 0.05). This could be highly affected by the lower protein content in samples leading to the weaker network development resulted in the higher leached starch molecules. Besides, higher amylose contents (developing the very rigid structure) in sample C, D, and E may cause the noodle strand to be easily breakable. In the case of colored rice noodles, amylose may not play an important role in noodle quality due to its low concentration, resulting in a higher cooking loss compared with white noodles. However, different protein and fiber contents among colored noodles could highly affect cooking loss. Protein can provide a stable structure because it develops networks through the interaction of amino acids (Ye & Sui, 2016), which contribute to lower cooking losses, whereas a higher fiber content commonly leads to poor texture properties of rice noodles due to lessening associations between starch-starch molecules or starch-protein/lipid molecules, which explain the highest value of cooking loss in colored rice noodle sample F (Table 2) (*p* < 0.05). *Tan, Li & Tan (2009)* described that some modified starches are applied in rice noodle manufacturing to improve the cooking qualities of the product, including decreasing cooking losses. As a result, different rice noodle formulas and ingredients can have an effect on cooking losses.

### Rehydration

The rehydration for white rice (Table 2) is lower compared to another study (Hormdok & Noomhorm, 2007 which reported the hydration in rice noodles was 194.15%–271.32%. However, it is in line with colored rice noodles. This study found that high rehydration resulted in a long cooking time in rice noodles. These are due to the high amounts of water required during cooking (Hatcher et al., 2002). High rehydration refers to the ability of the noodle strand to absorb water; a high value of this parameter commonly contributes to a soft or chewy texture of rice noodles. However, excessive rehydration could lead to properties of poor-quality rice noodles, such as high cooking loss and stickiness (Han, Cho & Koh, 2011). The highest rehydration values of sample E and F (*p* < 0.05) (Table 2) could be supported by the high contents of fiber, which increased water absorption (Huang & Lai, 2010). High protein content encourages high rehydration in rice noodles due to a high capacity to hold water or polar or charged molecules (Hsu et al., 2015). In contrast, low rehydration is due to a lower amount of water needed during cooking, which is more desirable of a rice noodle (*Wandee et al., 2015*).

### Pasting properties

Pasting properties are generally correlated with the quality of rice starch/flour that is used as a raw material for producing rice noodles (Hatcher et al., 2002). The pasting properties of white rice noodles (Table 3) are similar to those of rice starch: peak (2159–4240 cP), trough (1601–2698 cP), breakdown (502–2083 cP), final (1847–4908 cP), and setback viscosities (217–2141 cP) (Pinkaew et al., 2016). The variation of pasting properties in this research can also be influenced by the differences in proximate composition and amylose content (Huang & Lai, 2010). As the results presented, amylose and carbohydrate content of colored noodles are lower, with a higher crude fat, crude fiber and crude protein. So, lower in starch content or higher in crude fat, crude fiber and crude protein may result in reduction in peak viscosity. Moreover, interactions between the starch, fat, and protein of the blends may support that phenomenon.

High peak viscosity indicates a high capacity to absorb water and swelling of starch granules, providing a soft texture, increasing breakdown (Wang et al., 2016). High peak viscosity is encouraged by the high molecular weight of amylopectin (Hsu et al., 2015). High breakdown represents a low capacity of starch granules to resist shear forces, considered as low cooking intolerance (Chung, Cho & Lim, 2012). The high stability of rice noodle structure after cooling is indicated by a high final viscosity (Luo, Guo & Zhu, 2015). Good quality of rice noodles is reinforced by high setback due to the formation of a three-dimensional network formed by amylose (Otegbayo, Oguniyan & Akinwumi, 2013). A high capacity to re-associate starch molecules after cooking is attributed to the high setback, providing sturdiness to rice noodle structure (Yadav, Yadav & Kumar, 2011). However, starch or flour with higher setback tends to provide lower peak viscosity (Han, Cho & Koh, 2011). Hence, other starches (e.g., corn, tapioca, potato) can be added to noodle formulations to solve these problems (Wang et al., 2016).

### Texture properties

The low ability to break down the resistance of the noodle sample is indicated by a low tensile strength (breaking force) and extensibility, which is undesirable for rice noodles (Malahayati et al., 2017). Low tensile strength and extensibility are promoted by a weak noodle structure and low cooking tolerance (Nura et al., 2011). High amylose and protein contents are attributed to a high breaking force (Sandhu, Maninder & Mukesh, 2010). A strong structure is encouraged by three-dimensional network development of amylose (Han, Cho & Koh, 2011), which could also be reinforced by disulfide bond formation of amino acids from protein (Sangpring, Fukuoka & Ratanasumawong, 2015).

In general, higher values of hardness, cohesiveness, springiness, and chewiness, but lower values for adhesiveness and gumminess are indicative of a good-quality rice noodle (Hatcher et al., 2002). According to the results presented in Table 4, Sample E can be classified as the good white rice noodles and sample G for color rice noodles. Greater hardness in rice noodles is promoted by high amylose and protein contents. These are again due to the three-dimensional network of amylose (Ahmed, Qazi & Jamal, 2015). Moreover, amino acids in protein also form a network, increasing hardness (Chung, Cho & Lim, 2012). Lower cooking loss stimulates higher hardness in rice noodles by reducing the amount of solid components leached out during cooking (Luo, Guo & Zhu, 2015).

Adhesiveness refers to the stickiness of noodle texture; high stickiness is commonly undesirable in rice noodles (Pinkaew et al., 2016). According to the results (Table 4), sample D and sample F reached these criteria for white rice and color rice noodles, respectively. Higher hardness contributes to lower adhesiveness (Yadav, Yadav & Kumar, 2011). A longer duration of the steaming process leads to higher adhesiveness in the noodle texture (Huang & Lai, 2010). Cohesiveness is related to internal bonds that constitute the noodle structure. Normally, high cohesiveness and hardness result in high gumminess and chewiness due to the high energy required to break the noodle structure (Wu et al., 2015). Gumminess represents the energy required to disintegrate a semi-solid food until it is ready for human consumption and swallowing. However, high gumminess is not considered a required property (Sangpring, Fukuoka & Ratanasumawong, 2015). Springiness is the ability to return to an undeformed state after a compression force is removed; however, there was no difference in springiness among rice noodle samples (*p* < 0.05). Chewiness indicates the energy needed to chew solid food until it is ready for ingestion. High chewiness is considered a desirable property of rice noodles (Chou, Yen & Li, 2014). According to the textural properties, high tensile strength and extensibility of the noodles may result in greater acceptability.

### Sensory evaluation

The highest overall acceptability scores were found in sample B and sample H (Table 5), in the case of white and colored rice noodles (*p* < 0.05), respectively. A high acceptability of rice could be encouraged by higher scores for flavor, test, softness, and elasticity of the samples. On the other hand, higher values of texture properties (i.e., hardness, cohesiveness, gumminess, chewiness) resulted in lower acceptability scores in rice noodle samples. Furthermore, higher acceptability scores were promoted by higher values of pasting (i.e., setback final viscosities) and texture properties (i.e., tensile strength, extensibility) and lower cooking losses. Wang et al. (2016) and (Chou, Yen & Li, 2014) confirmed that the chemical composition, pasting, and cooking, as well as texture properties greatly affect rice noodle properties and acceptability.

The results of the Pearson’s correlation coefficient test also confirmed that the chemical composition as well as the pasting, cooking, and texture properties have an effect on rice noodle acceptance as shown in Table 6. Coefficient values can be in the range of +1 to -1, where +1 refers to a positive relationship, -1 points to a negative relationship, and 0 specifies that no relationship exists. Both negative and positive relationships between rice noodle acceptance and properties were found. Some components and properties, including crude fat, crude fiber, cooking loss, hardness, adhesiveness, etc., had a negative relationship with overall acceptability. However, only hardness showed a significant negative correlation with rice noodle acceptability (*p* < 0.05). On the other hand, carbohydrates, amylose, final viscosity, setback, tensile strength, and extensibility presented a positive relationship with rice noodle acceptance. The properties that had a significant positive correlation with acceptability were final and setback viscosity (*p* < 0.05).

**Table 6:** Pearson correlation test between acceptability and properties of rice noodles.

Chemical composition	Coefficient value
Crude fat	−0.527
Crude fiber	−0.364
Crude protein	−0.260
Carbohydrate	0.405
Amylose content	0.226
Pasting properties of rice noodle powder	Coefficient value
Peak viscosity	0.661
Trough viscosity	0.661
Breakdown viscosity	0.567
Final viscosity	0.777^*^
Setback viscosity	0.782^*^
Cooking properties of rice noodles	Coefficient value
Cooking time	−0.619
Cooking loss	−0.453
Rehydration	−0.614
Texture properties of rice noodles	Coefficient value
Tensile strength	0.387
Extensibility	0.072
Hardness	−0.758^*^
Adhesiveness	−0.551
Cohesiveness	−0.264
Gumminess	−0.523
Springiness	−0.196
Chewiness	−0.501

**Notes.**

* Correlation is significant at 0.05 level (2-tailed). Coefficient values; +1 = a positive relationship, −1 = a negative relationship, and 0 = no relationship exists.

## Conclusions

Eight commercial rice noodles that are readily available in Thai markets (A, B, C, D, F, G, and H) had different quality attributes, including color attributes, cooking properties, pasting properties, texture properties, and sensory scores. The acceptability scores of all noodle samples were in the range of 5.30–7.00; sample B had the highest acceptance score (*p* < 0.05).

Greater acceptability of the rice noodle could be initiated by desirable texture properties (high tensile strength and extensibility), required pasting properties (high setback and final viscosity), lower cooking loss, suitable amylose content, and higher protein content. Whereas the lower acceptability of other rice noodles could be caused by their undesirable properties, such as poor texture (low tensile strength and extensibility and excessively high hardness, stickiness, gumminess, and chewiness) and cooking properties (high cooking loss and too high rehydration) due to the lower amylose and higher fiber content.

##  Supplemental Information

10.7717/peerj.11113/supp-1 Supplemental Information 1 Raw data

## Additional Information and Declarations

### Data Availability

The following information was supplied regarding data availability: Raw data are available as a Supplementary File.
